# PalmXplore: oil palm gene database

**DOI:** 10.1093/database/bay095

**Published:** 2018-09-18

**Authors:** Nik Shazana Nik Mohd Sanusi, Rozana Rosli, Mohd Amin Ab Halim, Kuang-Lim Chan, Jayanthi Nagappan, Norazah Azizi, Nadzirah Amiruddin, Tatiana V Tatarinova, Eng-Ti Leslie Low

**Affiliations:** 1 Advanced Biotechnology and Breeding Centre, Malaysian Palm Oil Board, No. 6, Persiaran Institusi, Bandar Baru Bangi, Kajang, Selangor, Malaysia; 2 Genomics and Computational Biology Research Group, University of South Wales,Pontypridd, Wales, UK; 3 Department of Biology, University of La Verne,1950 Third Street La Verne, CA, USA

## Abstract

A set of *Elaeis guineensis* genes had been generated by combining two gene prediction pipelines: Fgenesh++ developed by Softberry and Seqping by the Malaysian Palm Oil Board. PalmXplore was developed to provide a scalable data repository and a user-friendly search engine system to efficiently store, manage and retrieve the oil palm gene sequences and annotations. Information deposited in PalmXplore includes predicted genes, their genomic coordinates, as well as the annotations derived from external databases, such as Pfam, Gene Ontology and Kyoto Encyclopedia of Genes and Genomes. Information about genes related to important traits, such as those involved in fatty acid biosynthesis (FAB) and disease resistance, is also provided. The system offers Basic Local Alignment Search Tool homology search, where the results can be downloaded or visualized in the oil palm genome browser (MYPalmViewer). PalmXplore is regularly updated offering new features, improvements to genome annotation and new genomic sequences. The system is freely accessible at http://palmxplore.mpob.gov.my.

## Introduction

Oil palm is a major source of oil that has become a common ingredient in many consumer products and industrial applications, such as biofuel, a renewable alternative to petroleum. The oil palm is globally important and the highest yielding oil-bearing crop in the world, with an average national yield of 4 tonnes of oil per hectare per year. Its production has increased over the decades with a world production of 69.89 million metric tons in 2017–18, which is an increase of about 7.4% from 2016–17 (1). Palm oil dominates the global market, contributing up to 55% of the total world exports of oils and fats (2). The upstream subsectors of oil palm industry particularly in genomics-based technologies have gone through tremendous transformation over the past few decades (3). The importance of the oil palm inspired the Malaysian Palm Oil Board (MPOB) to sequence and assemble the genome of two oil palm species, *Elaeis guineensis* and *Elaeis oleifera* to further improve the industry (4). A breakthrough achievement by MPOB was the discovery of the *SHELL* gene that is responsible for the dura (thick-shell), pisifera (shell-less) and tenera (thin-shell) fruit forms (5), which have a significant impact on yield. Following this, Singh *et al.* (2014) successfully identified the *VIRESCENS* gene that controls the exocarp colour of the oil palm fruit (6). Another major success was the identification of the epimutation in the *MANTLED* gene that causes the mantled somaclonal abnormality in oil palm that often results in bunch failure and drastic yield reduction (7).

To address the growing demand for exploring and retrieving oil palm data, a web portal for the oil palm genome information was developed (Genomsawit portal: http://genomsawit.mpob.gov.my) (8). This portal was designed as an initial access point for oil palm web-based information systems and specialized datasets. When the oil palm genome sequence was published in 2013, the assembled sequences represented about 83% of the 1.8 Gb genome sequence (4). At that time, only a draft gene model prediction was available. Therefore, in order to improve the accuracy of gene model prediction and subsequent annotation of the genome, gene models from two pipelines namely Fgenesh++ (9) and an in-house tool-termed Seqping (10) were integrated (11). By using these pipelines, we were able to predict a total of 26 059 high-quality and validated gene models in the *E. guineensis* genome. Genes associated with imperative agronomic traits, such as fatty acid biosynthesis (FAB) and disease resistance, were also identified (11). In order to assist in the use of these datasets, we have developed a searchable database and information system called PalmXplore (http://palmxplore.mpob.gov.my). PalmXplore features a series of user-friendly search engines, information browsers and interactive visualization tools for accessing the oil palm gene information and associated functional annotations.

The portal uses the information from and is linked to the Enzyme Code from Kyoto Encyclopedia of Genes and Genomes (KEGG) (12), Gene Ontology (GO) (13) and Pfam version 29.0 (14) databases. User-friendly query interfaces and bioinformatics tools such as Basic Local Alignment Search Tool (BLAST) (15) and an oil palm genome browser called MYPalmViewer (16, 17) have been developed within the system to help users in deciphering important biological information from the datasets.

PalmXplore is able to efficiently handle the oil palm genome data and is scalable to keep up with data growth. It can also provide necessary input/output operations to submit selected entries to the integrated bioinformatics analytics toolkit. The primary genomic data types available include predicted oil palm genes and assembled scaffold information, DNA and protein sequences and functional annotation results. Integrating these diverse data types in an online user-friendly database that is easy to query, view and download was essential to maximize utility of these valuable research data.

In summary, PalmXplore provides the following features:

- Representative oil palm gene models
- Oil palm genome Pisifera5-build (P5-build)
- Basic and advanced search functionalities
- Oil palm gene identifiers, coding sequence ID (CDS ID) and P5-build assembled scaffolds (Scaffold ID) browsers
- Integration to the Genomsawit portal
- Integrated bioinformatics tools and external databases:
  – BLAST
  – Oil palm genome browser (MYPalmViewer)
  – GO
  - Enzyme Code from KEGG
  – Pfam

## Materials and methods

### Source of the oil palm gene data

Genic regions of the genome were predicted by integrating the gene models of two pipelines, the established Fgenesh++ (9) and an in-house tool, Seqping (10, 11). Two approaches were taken to predict good quality homologous proteins in oil palm. The first uses P5-build genomic scaffolds of an *AVROS* (Algemene Vereniging van Rubberplanterter Oostkust van Sumatra) *pisifera* palm (4) and known proteins from closely related organisms such as the date palm, as reference sequences for the Fgenesh++ pipeline (with generic parameters for monocots) to identify a set of predicted oil palm gene models encoding highly homologous proteins. Gene models with significant BLAST hit (E-value cut-off: e^−10^) to known plant proteins from the NCBI non-redundant (NR) database were used as a training set for the Fgenesh++ pipeline to develop oil palm Hidden Markov Model (HMM). The HMM was used to identify the genic regions in the oil palm genome sequence. Subsequently, BLAST 2 Sequences was executed to compare the predicted gene models to the protein sequences from the plant NR database. The cut-offs were percent identity ≥ 50, score ≥ 100, coverage of predicted protein ≥ 80% and coverage of homologous protein ≥ 80%. Sequence similarity search between the predicted genes and the *E. guineensis* mRNA dataset (5, 18, 19, 20, 21) with an identity cut-off of ≥ 90% was also carried out. A total of 27 915 Fgenesh++ gene models had notable similarities to the *E. guineensis* mRNA dataset and RefSeq proteins (22). The in-house-developed gene prediction pipeline, Seqping, was used in parallel as a second approach to validate and subsequently improve the accuracy of the genes predicted by Fgenesh++. The self-trained HMM was used to make gene predictions by incorporating the transcriptomic datasets of oil palm. Here, the pipeline processed the genome and transcriptome sequences using GlimmerHMM version 3.0 (23, 24), SNAP (25) and AUGUSTUS version 2.6.1 (26) pipelines, followed by MAKER2 (27) program to combine the predictions from the three tools in association with the transcriptomic evidence. The predicted sequences were compared to RefSeq protein sequences and oil palm transcriptome dataset via BLASTX (E-value cut-off: e^−10^), resulting in 17 680 predicted genes with significant similarities. Gene models predicted using the two approaches were unified, resulting in 26 059 high-quality genes (11).

### Database and web interface implementation

The PalmXplore system consists of two major components: a database to store and administer the data and a high-level web interface. The back end of this system was organized with a relational model and stored in the MySQL (https://www.mysql.com/) database management system. phpMyAdmin (https://www.phpmyadmin.net/) and mysql-workbench 6.3 (https://www.mysql.com) were used for data modelling and database development and administration. The web interfaces were constructed using PHP scripting language, HTML5, CSS3 styling codes and JavaScript and operate on the Apache web server. It was designed and tested for web browsers and derived rendering engines (Windows operating system (OS): Firefox 10.0 and higher, Google Chrome 21.0 and higher, Safari 5; Linux OS: Firefox 3.6 and higher, Google Chrome 37.0 and higher, Opera 12.0 and higher; Mac OS X version: Firefox 30.0 and higher, Google Chrome 41.0 and higher, Safari 9.1). The development of the front end was facilitated and empowered by Bootstrap3 and AngularJS frameworks (http://getbootstrap.com/) to address the balance between design and implementation. The system sites were also developed to be mobile friendly. They were optimized for responsiveness on client systems regardless of the sizes of device used, whereby the fluid grid system implemented was scalable to 12 columns as the device or viewport size increased. The overall performance of the web and system was analysed by Yslow (http://yslow.org), based on predefined rule set identified by Yahoo!. Google Analytics (https://www.google.com/analytics) is currently used to track and report website traffic and user activities.

### PalmXplore system architecture and database design

**Figure 1:**
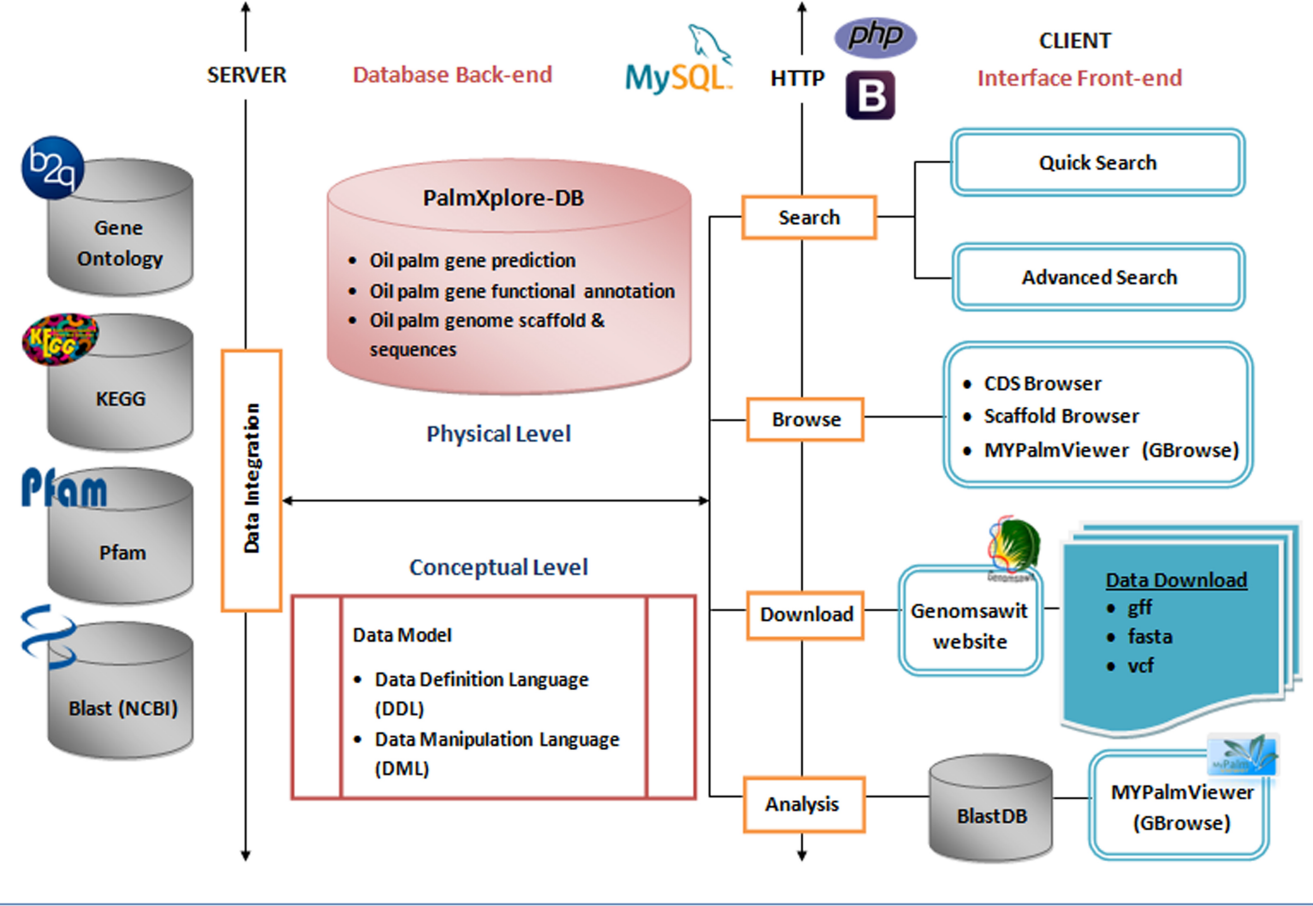
Overview of the PalmXplore system architecture. The system architecture is based on the client/server architecture. The PalmXplore-DB contains a list of predicted oil palm genes, functional annotations of the genes with integrative access to external databases and oil palm genome scaffolds and sequences.

A three-tiered client/server structural design was implemented with this system (28), through which the presentation (front end), processing (conceptual) and data storage (physical) are logically divided. Here, the conceptual level plays the crucial role in streamlining the integration, sharing and exchange of available data (Figure 1). The system’s architecture makes the existing modules easy to maintain and is scalable and upgradable, without the need to redesign the database scheme and storage properties for new data.

**Figure 2:**
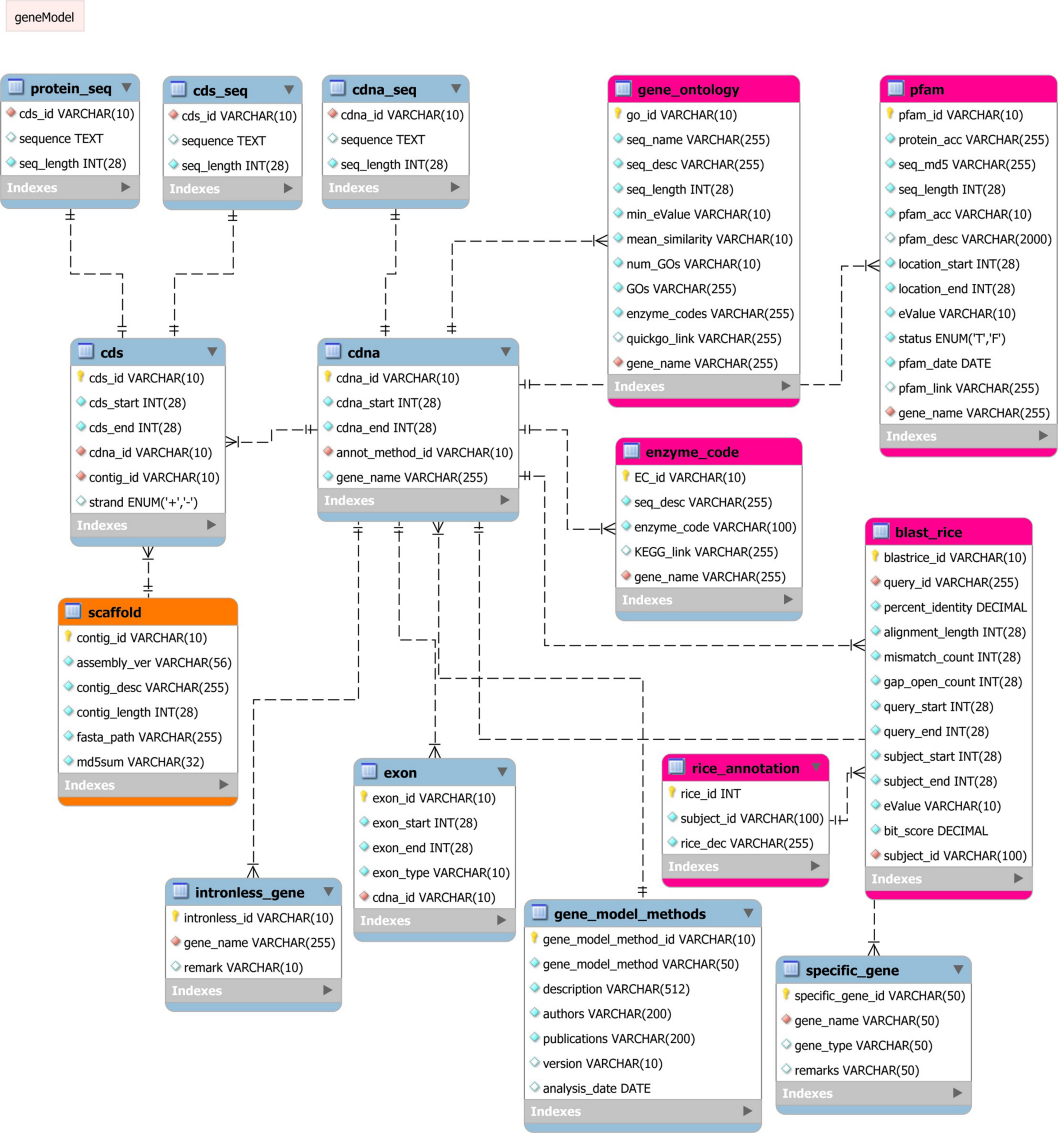
ERD of PalmXplore-DB. The ERD shows the conceptual data structure used in PalmXplore-DB. Entities and relationships are represented as boxes and dotted lines between the boxes, respectively. The database structure consists of 15 tables presented in three modules (represented by different colours).

The entire module was modelled into a relational database management system (RDBMS). PalmXplore-DB consists of sequence data and annotations derived from different annotation methods. These data need to be stored, indexed and searched efficiently. RDBMS uses indexes to sort data and link information from different tables through the use of foreign keys. Other tables may refer to that foreign key, so as to create a link between their data pieces. This comes in handy for applications that are heavy on data analysis and thus, is a good choice for genome or gene annotation databases (29). General performance of SQL queries was checked using the EXPLAIN command in MySQL (Supplementary file 1). In Figure 1, a detailed schema of the PalmXplore-DB is illustrated. Here, the conceptual structure of this database was visualized in the form of an Entity-Relationship Diagram (ERD; Figure 2). Three modules of closely linked relations or tables of the database were created. In the first module, nine entities (protein_sequence, cds_sequence, cdna_sequence, cds, cdna, intronless_gene, exon, gene_model_methods and specific_gene) were constructed whereby, they primarily stored the core information on oil palm protein-coding genes (11). The second module was designed with five entities that manages data on functional annotation of oil palm genes from KEGG (12), GO (13) and Pfam (14) databases. Moreover, additional information on annotation that resulted from protein sequence comparison of oil palm and rice (*Oryza sativa*) by NCBI Protein BLAST (30) were included in this database. The third module is a single table of Scaffold ID.

## Results

**Figure 3:**
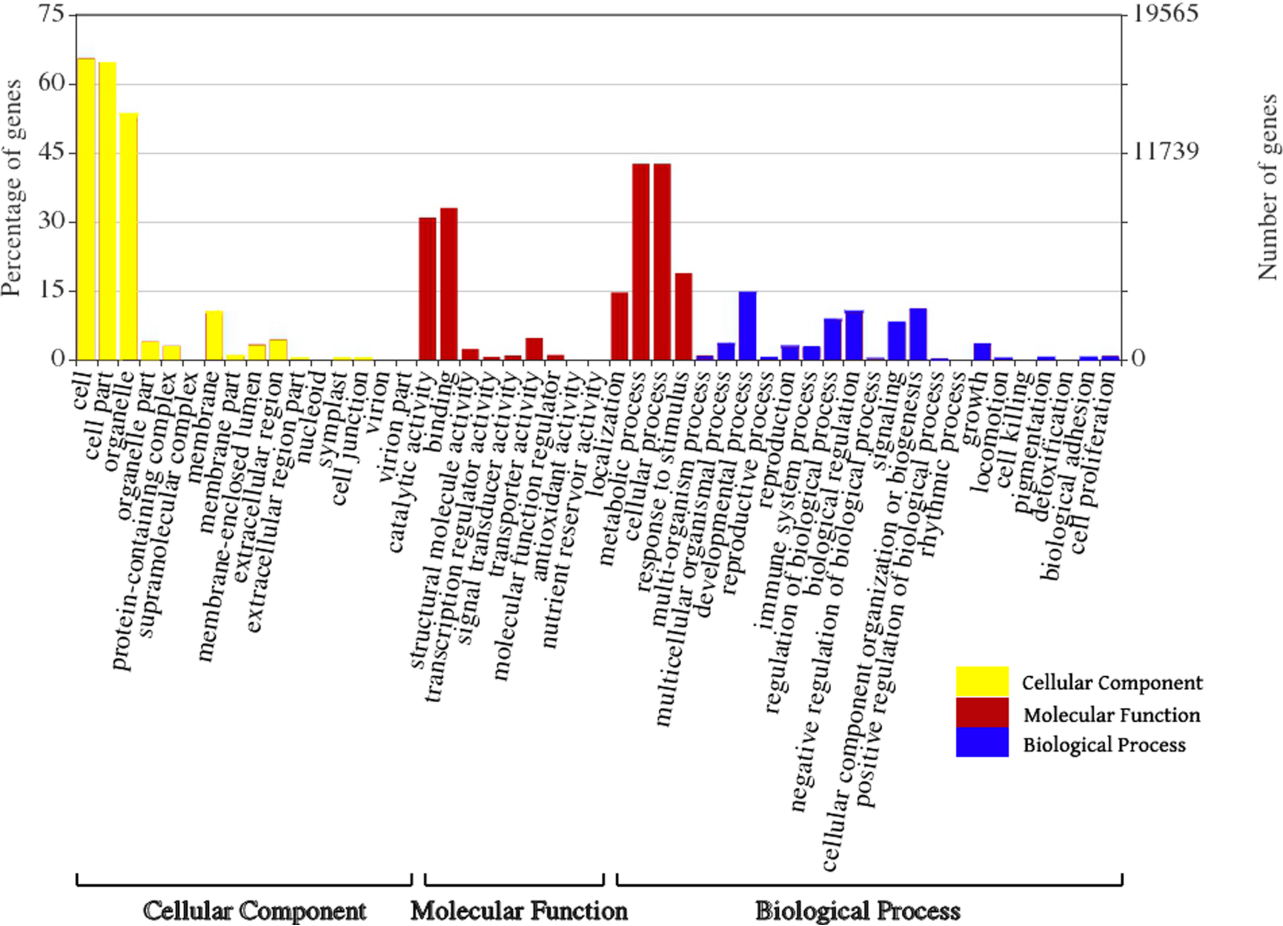
Oil palm genes classification based on GO annotation.

The improved predictions were important for a well-defined annotation of the oil palm genes. Hence, two methods were tested in acquiring high-quality gene sets for oil palm. In the first prediction with Fgenesh++, ∼27 915 genes that had similarity to the oil palm transcriptome dataset were predicted. The in-house-developed gene prediction pipeline Seqping predicted ∼17 680 genes that had significant similarities to the oil palm transcriptome dataset. Combining the results of the two pipelines produced a high-quality set of 26 059 genes that were subsequently imported into the PalmXplore database. Figure 3 shows the GO functional classifications of the oil palm genes (31).

### Database components

In this database (PalmXplore-DB), relevant information pertaining to the oil palm genome had been deposited. The most recent was the* E. guineensis* P5-build and a collection of predicted oil palm genes. The unified data set of 26 059 genes from Fgenesh++ and Seqping was deposited into the database. Within these high-quality representative oil palm gene models, 3672 (14.1%) genes were identified to be intronless, 42 were classified as FAB genes and 210 were identified as resistance genes (11). The published *SHELL* (5), *VIRESCENS* (6) and *MANTLED* (7) genes were also included in this database. Table 1 shows a summary of the data deposited in the PalmXplore system. 

**Table 1:** Summary of the data deposited in PalmXplore system

**P5-build oil palm genome statistics**			
Total number of scaffolds	40 360		
Average / N50 / largest scaffold sizes (bp)	38 036 / 1 045 414 / 22 100 610		
Number of bases (bp)	1 535 150 282		
**Oil palm gene models**			
	Representative	Fgenesh++	Seqping
Number of genes	26 059	27 915	17 680
Average length (bp)	1 239	1 120	1 193
Gene density (gene/Mb)	16.98	18.19	11.52
Average exon per gene	5.4	5.1	6.0
Average exon length (bp)	252	237	197
Number of genes annotated to GO term(s)	21 572	-	-
Number of genes with Enzyme Code (KEGG)	6 195	-	-
**Others**			
Intronless genes	3 658		
Resistance (R) genes	210		
FAB genes	42		

### Data access and retrieval

**Figure 4:**
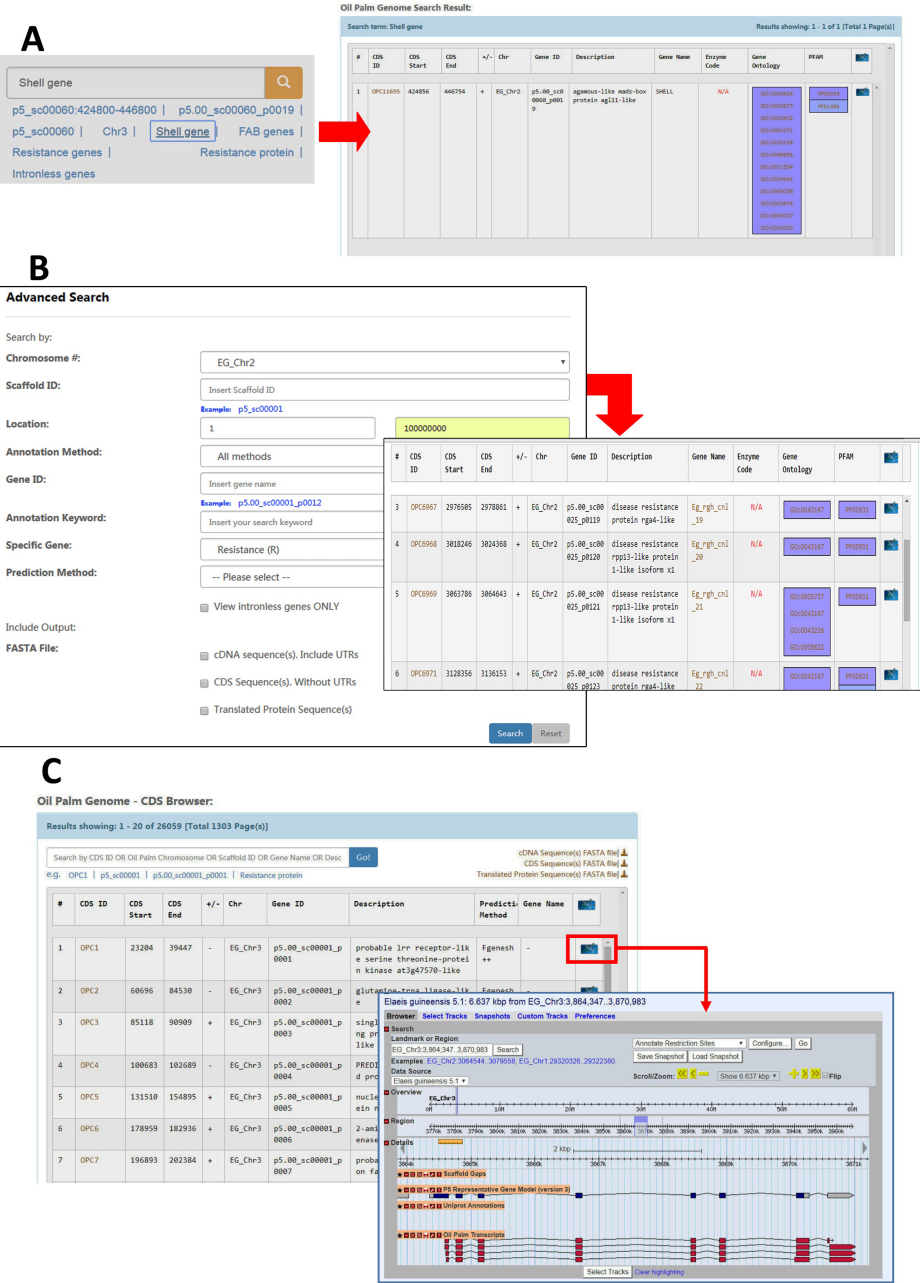
Search and browse options in the PalmXplore system**.** (**A**) Basic search: search oil palm genes by Gene ID, Scaffold ID, chromosome number, keywords, specific gene or location on the genome. (**B**) Advanced search: refine search results by entering multiple options. (**C**) MYPalmViewer: visualize and navigate searched gene in the oil palm genome sequence. Annotation data is also available. (**D**) CDS browser: browse the list of predicted genes.

PalmXplore has made the access and retrieval of data feasible, and the user-friendly web interface facilitates efficient and comprehensive search and browsing of the predicted oil palm (EG) genes. A simple free-text search of the genes is available. PalmXplore’s record identifiers and keywords can also be used in the search. These include CDS ID, Scaffold ID, oil palm chromosome, gene name and gene annotation. Additionally, searches on specific genes, such as intronless, fatty acid biosynthetic, disease-resistance, *SHELL*, *VIRESCENS* and *MANTLED* genes can be easily performed (Figure 4A).

In the advanced search filter, a combination of search criteria will assist users in applying greater control over how the customized search operates on the oil palm gene models. The PalmXplore database was designed with the integration of public databases, such as KEGG, GO and Pfam to provide an in-depth description of the oil palm predicted genes and its functional annotation. The database also permits users to search for a gene by its location on the genome, ID or putative function (e.g. hydrolase or protein kinase) (Figure 4B).

The search output includes the sequence data used, prediction pipelines, functional annotation, genome position and a link to MYPalmViewer (Figure 4C). Additional information to cross-reference the prediction with other databases (KEGG, GO and Pfam) is also available. The search results can be exported for further analysis. The data could also be retrieved by browsing the list of CDS ID and Scaffold ID in the form of populated tables (Figure 4D).

### System interoperability

**Figure 5:**
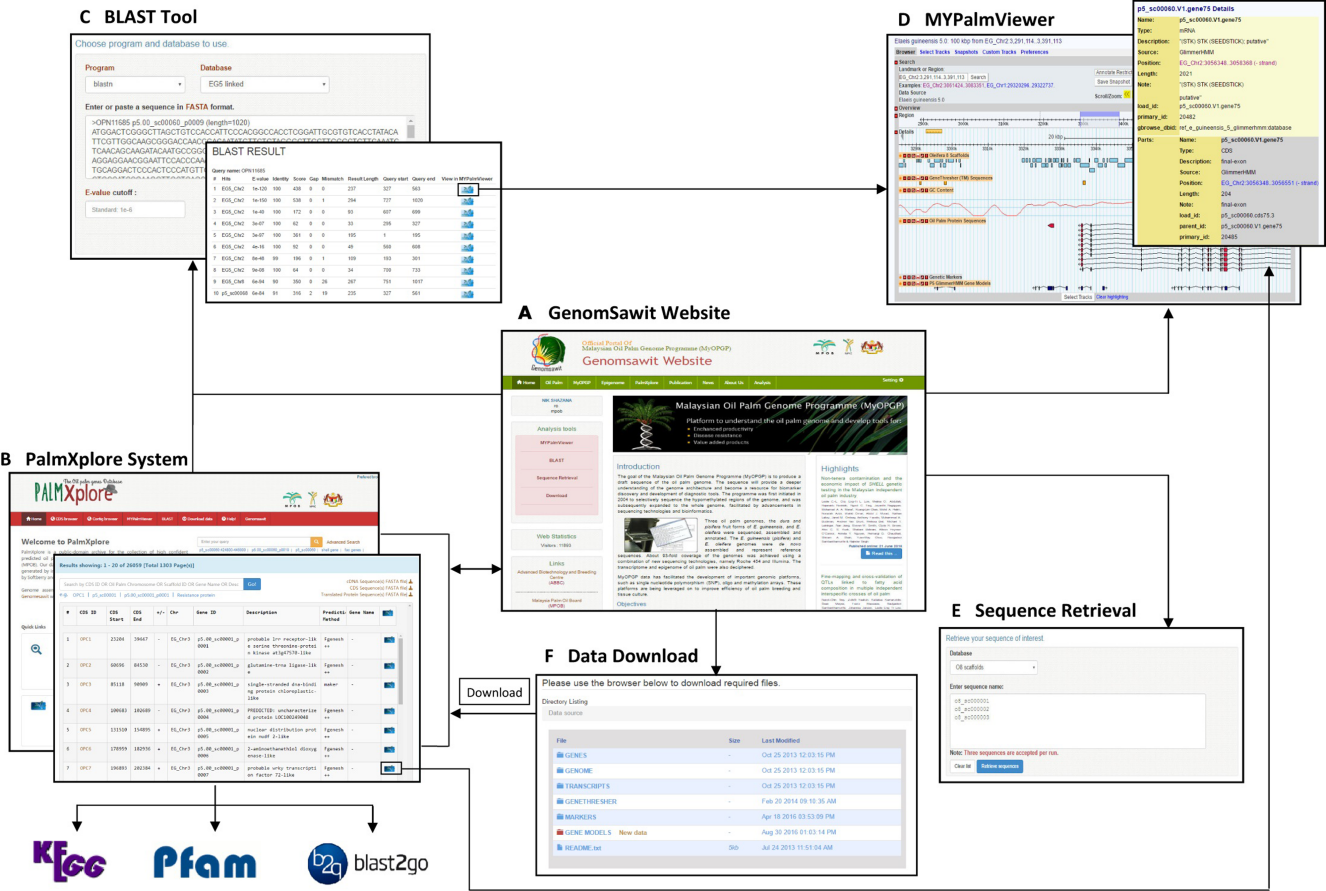
Interoperability with web portal and Bioinformatics analysis tools. (**A**) Genomsawit portal: a web portal for the oil palm genome information. Genome assemblies and gene models are available for download here. (**B**) PalmXplore system manages oil palm gene data deposition and queries. (**C**) BLAST as an alignment tool. (**D**) MYPalmViewer to visualise oil palm genomes, genes, genetic markers and others; (**E**) sequence retrieval of oil palm genomic data; and (**F**) data download facility.

PalmXplore system is interoperable with the Genomsawit portal and bioinformatics analysis tools such as the BLAST program and genome browser (Figure 5). Genomsawit (Figure 5A) is a web portal, which provides comprehensive, updated and free oil palm genome information. Currently, Genomsawit provides the genome data for *E. guineensis* and *E. oleifera* (4), gene models (4, 11), transcripts (4), markers (32, 33) and GeneThresher data (34).

Sequence similarity search function can be done using BLAST (Figure 5C), where reference databases were prepared using the NCBI’s makeblastdb tool. Twelve databases containing genomic, transcriptomic, gene annotation and GeneThresher data of *E. guineensis* and *E. oleifera* are readily available for nucleotide or protein sequence search.

MYPalmViewer (Figure 5D) is embedded into the portal and can be accessed through the search result page of predicted genes or by directly clicking on the link available on the menu tab. The BLAST results are also linked to sequence objects in MYPalmViewer. The hits are visualized as features in the overview, region and detail panels of MYPalmViewer. The features in MYPalmViewer are hyperlinked to a page with additional information and sequences, as well as to external databases. MYPalmViewer allows all tracks to be displayed on the same view concurrently. The tracks are customizable; color, shape, size and position on the display are all user-configurable. Apart from that, users are allowed to upload custom track data in a variety of file formats [BED, GBrowse Feature File Format, GFF, GFF3, Wiggle (WIG), BAM and SAM]. In addition to genome browsing, MYPalmViewer offers several other capabilities, such as search engines, detailed view pages for each gene, interactive genome navigation and download functions.

## Conclusion

When the oil palm genome sequence was published in 2013, a resource was needed to host the oil palm sequences; hence, the Genomsawit portal was framed out. Later, with the availability of the high-quality predicted genes, PalmXplore was created as an appendage to the Genomsawit portal. PalmXplore is the first publicly available gene resource depository and search engine for MPOB’s oil palm genome data. With the accessibility of this system, it facilitates proficient and comprehensive search and browsing of the sequence information and annotations of oil palm genes. Moreover, this system has been integrated with the BLAST search options and the results can be visualized via the oil palm genome browser (MYPalmViewer). Apart from that, the information in PalmXplore provides fundamental and important information needed to expedite biological research pertaining to oil palm. The information in this database further aids in identification of new genes and gene families that will be responsible for traits of interest such as the height, FAB and disease resistance genes (35). The database is continuously updated with new features, improvements to the oil palm genome and gene models, as they become available, along with the associated data mining and updated versions of bioinformatics analysis tools. PalmXplore is freely accessible at http://palmxplore.mpob.gov.my.

## Supplementary Material

Supplementary Data

## Notes

**Database URL:** http://palmxplore.mpob.gov.my
