# Supplementary material for: PalmXplore: oil palm gene database
Source: Database (Oxford). 2018 Sep 18;2018:bay095. doi: 10.1093/database/bay095 (PMC6146135; doi:10.1093/database/bay095)
Supplement: Supplementary Data [file bay095_supp.zip › Supplementary_1.pdf]

Query Sanity Checks with MySQL EXPLAIN

1) System Search:

example -

```
mysql> EXPLAIN  select DISTINCT cds.cds_id, cds.contig_id, cdna.cdna_name, cdna.cdna_id, exon.exon_start, exon.exon_end, enzyme_code_link.seq_desc, pfam.pfam_acc, pfam.
pfam_desc, blast2go.seq_desc,  gene_name.old_name, gene_name.new_name FROM cds JOIN cdna ON cds.cdna_id=cdna.cdna_id JOIN exon ON cdna.cdna_id=exon.cdna_id JOIN gene_na
me ON cdna.cdna_name=gene_name.old_name LEFT JOIN enzyme_code_link ON cdna.cdna_name=enzyme_code_link.gene_name LEFT JOIN pfam ON cdna.cdna_name=pfam.gene_name LEFT JOI
N blast2go ON cdna.cdna_name=blast2go.gene_name WHERE ((cds.contig_id = 'p5_sc00060') AND ((exon.exon_start >= '1' AND exon.exon_start <= '1000000000') OR (exon.exon_en
d >= '1' AND exon.exon_end <= '1000000000'))) GROUP BY cds.cds_id;
+----+-------------+------------------+--------+--------------------------------------+--------------------+---------+----------------------------+------+---------------
-------------------------------+
| id | select_type | table            | type   | possible_keys                        | key                | key_len | ref                        | rows | Extra
                               |
+----+-------------+------------------+--------+--------------------------------------+--------------------+---------+----------------------------+------+---------------
-------------------------------+
|  1 | SIMPLE      | cds              | ref    | fk_cds_contig1_idx,fk_cds_cdna1_idx | fk_cds_contig1_idx | 32      | const                      |   61 | Using where; U
sing temporary; Using filesort |
|  1 | SIMPLE      | cdna             | eq_ref | PRIMARY,cdna_id,cdna_name            | PRIMARY            | 32      | genemodelv2.cds.cdna_id    |    1 |
                               |
|  1 | SIMPLE      | enzyme_code_link | ref    | gene_name                            | gene_name          | 767     | genemodelv2.cdna.cdna_name |    1 |
                               |
|  1 | SIMPLE      | gene_name        | ref    | old_name                             | old_name           | 767     | genemodelv2.cdna.cdna_name |    1 |
                               |
|  1 | SIMPLE      | pfam             | ref    | gene_name                            | gene_name          | 767     | genemodelv2.cdna.cdna_name |    1 |
                               |
|  1 | SIMPLE      | blast2go         | ref    | gene_name                            | gene_name          | 767     | genemodelv2.cdna.cdna_name |    1 |
                               |
|  1 | SIMPLE      | exon             | ref    | fk_exon_cdna1_idx                    | fk_exon_cdna1_idx  | 32      | genemodelv2.cds.cdna_id    |    5 | Using where
                               |
+----+-------------+------------------+--------+--------------------------------------+--------------------+---------+----------------------------+------+---------------
-------------------------------+
7 rows in set (0.00 sec)
```

Select statement:

```
mysql> select DISTINCT cds.cds_id, cds.contig_id, cdna.cdna_id, cdna.cdna_name, cdna.annot_method_id, exon.exon_start, exon.exon_end,enzyme_code_link.gene_name, enzyme_
code_link.enzyme_code, enzyme_code_link.seq_desc, blast2go.gene_name, blast2go.GOs, blast2go.seq_desc, pfam.gene_name, pfam.pfam_acc, pfam.pfam_desc,  gene_name.old_nam
e, gene_name.new_name, intronless.gene_name, gbrowse.location FROM cds JOIN cdna ON cds.cdna_id=cdna.cdna_id JOIN gbrowse ON cdna.cdna_id=gbrowse.cdna_id JOIN exon ON c
dna.cdna_id=exon.cdna_id  JOIN gene_name ON cdna.cdna_name=gene_name.old_name LEFT JOIN enzyme_code_link ON cdna.cdna_name=enzyme_code_link.gene_name LEFT JOIN pfam ON
cdna.cdna_name=pfam.gene_name LEFT JOIN blast2go ON cdna.cdna_name=blast2go.gene_name JOIN intronless ON cdna.cdna_name=intronless.gene_name WHERE (((cds.contig_id = 'p
5_sc00060') AND ((exon.exon_start >= '1' AND exon.exon_start <= '1000000000') OR (exon.exon_end >= '1' AND exon.exon_end <= '1000000000')) )) GROUP BY cds.cds_id;
                                                                                                                                                      | PREDICTED: uncharacte
rized protein LOC100822943                   | p5_sc00060_gene_215                                  | PF04504   | Protein of unknown function, DUF573
  | p5_sc00060_gene_215                      | p5.00_sc00060_p0104 | p5_sc00060_gene_215                             | EG_Chr2:1321485..1323167 |
| OPC11784 | p5_sc00060 | OPN11784 | p5_sc00060_gene_226                                  | Fgenesh++        |     2350586 |   2351305 | NULL                 | NULL
                              | NULL                                                                    | p5_sc00060_gene_226                             | F:DNA binding; F:mole
cular_function
                                                                                                                                                      | agamous-like mads-box
 protein agl80-like                          | p5_sc00060_gene_226                                  | PF00319   | SRF-type transcription factor (DNA-binding and dimerisation domain
) | p5_sc00060_gene_226                      | p5.00_sc00060_p0108 | p5_sc00060_gene_226                             | EG_Chr2:1151797..1153750 |
| OPC11800 | p5_sc00060 | OPN11800 | snap_masked-p5_sc00060-processed-gene-27.26     | maker            |     2750128 |   2750487 | NULL                 | NULL
                              | NULL                                                                    | snap_masked-p5_sc00060-processed-gene-27.26     | -

                                                                                                                                                      | predicted protein
                                             | NULL                                                 | NULL      | NULL
  | snap_masked-p5_sc00060-processed-gene-27.26     | p5.00_sc00060_p0124 | snap_masked-p5_sc00060-processed-gene-27.26     | EG_Chr2:752615..752974   |
| OPC11816 | p5_sc00060 | OPN11816 | p5_sc00060_gene_312                                  | Fgenesh++        |     3339781 |   3340827 | p5_sc00060_gene_312 | EC:4.2.1.51; EC:4
.2.1.91; EC:5.4.99.5          | arogenate dehydratase prephenate dehydratase chloroplastic-like | p5_sc00060_gene_312                             | F:lyase activity; P:c
ellular amino acid metabolic process; P:biosynthetic process; C:plastid; F:isomerase activity
                                                                                                                                                      | arogenate dehydratase
 prephenate dehydratase chloroplastic-like | p5_sc00060_gene_312                                  | PF00800   | Prephenate dehydratase
  | p5_sc00060_gene_312                      | p5.00_sc00060_p0140 | p5_sc00060_gene_312                             | EG_Chr2:162275..163808   |
| OPC11818 | p5_sc00060 | OPN11818 | p5_sc00060_gene_326                                  | Fgenesh++        |     3498003 |   3498959 | p5_sc00060_gene_326 | EC:4.3.3.6
                              | probable pyridoxal biosynthesis protein pdx1-like               | p5_sc00060_gene_326                             | P:biological_process;
 F:lyase activity; P:cellular amino acid metabolic process; P:biosynthetic process; C:plasma membrane; F:molecular_function; P:response to stress; P:small molecule meta
bolic process; C:cell; P:carbohydrate metabolic process; P:cofactor metabolic process; C:cytosol; P:cellular nitrogen compound metabolic process | probable pyridoxal bi
osynthesis protein pdx1-like                 | p5_sc00060_gene_326                                  | PF01680   | SOR/SNZ family
  | p5_sc00060_gene_326                      | p5.00_sc00060_p0142 | p5_sc00060_gene_326                             | EG_Chr2:3954..5099       |
+----------+------------+----------+--------------------------------------------------+------------------+------------+----------+---------------------+-----------------
-----------------------------+-----------------------------------------------------------------+-----------------------------------------------------+--------------------
----------------------------------------------------------------------------------------------------------------------------------------------------------------------
----------------------------------------------------------------------------------------------------------------------------------------------------+--------------------
---------------------------------------------+---------------------------------------------------+----------+---------------------------------------------------------
--+-----------------------------------------------+----------------------+-------------------------------------------------+-------------------------+
15 rows in set (1.22 sec)
```

2) CDS Browser:

```
mysql> EXPLAIN select DISTINCT cds.cds_id, cds.cds_start, cds.cds_end, cds.strand, cds.contig_id, cds.cdna_id, cdna.cdna_id, cdna.cdna_name, cdna.cdna_start, cdna.cdna_
end, cdna.annot_method_id, blast2go.gene_name, blast2go.seq_desc, gene_name.old_name, gene_name.new_name FROM cds JOIN cdna ON cds.cdna_id=cdna.cdna_id JOIN blast2go ON
 cdna.cdna_name=blast2go.gene_name JOIN gene_name ON cdna.cdna_name=gene_name.old_name ORDER BY CAST(MID(cds.cds_id,4) AS UNSIGNED);
+----+-------------+-----------+--------+----------------------------+-----------+---------+-----------------------------+-------+---------------------------------+
| id | select_type | table     | type   | possible_keys              | key       | key_len | ref                         | rows  | Extra                           |
+----+-------------+-----------+--------+----------------------------+-----------+---------+-----------------------------+-------+---------------------------------+
|  1 | SIMPLE      | cds       | index  | fk_cds_cdna1_idx           | NULL      | 32      | NULL                        | 26059 | Using temporary; Using filesort |
|  1 | SIMPLE      | cdna      | eq_ref | PRIMARY,cdna_id,cdna_name  | PRIMARY   | 32      | genemodelv2.cds.cdna_id     |     1 |                                 |
|  1 | SIMPLE      | gene_name | ref    | old_name                   | old_name  | 767     | genemodelv2.cdna.cdna_name  |     1 |                                 |
|  1 | SIMPLE      | blast2go  | ref    | gene_name                  | gene_name | 767     | genemodelv2.cdna.cdna_name  |     1 |                                 |
+----+-------------+-----------+--------+----------------------------+-----------+---------+-----------------------------+-------+---------------------------------+
4 rows in set (0.00 sec)
```

Select statement:

```
mysql> select DISTINCT cds.cds_id, cds.cds_start, cds.cds_end, cds.strand, cds.contig_id, cds.cdna_id, cdna.cdna_id, cdna.cdna_name, cdna.cdna_start, cdna.cdna_end, cdn
a.annot_method_id, blast2go.gene_name, blast2go.seq_desc, gbrowse.location, gene_name.old_name, gene_name.new_name FROM cds JOIN cdna ON cds.cdna_id=cdna.cdna_id JOIN b
last2go ON cdna.cdna_name=blast2go.gene_name JOIN gbrowse ON cdna.cdna_id=gbrowse.cdna_id JOIN gene_name ON cdna.cdna_name=gene_name.old_name ORDER BY CAST(MID(cds.cds_
id,4) AS UNSIGNED);

| OPC26053 |       120 |      1712 | -      | p5_sc39959 | OPN26053 | OPN26053 | p5_sc39959_gene_1                                      |        120 |      1712 | Fgenesh++
   | p5_sc39959_gene_1                                  | g-type lectin s-receptor-like serine threonine-protein kinase rlk1-like
     | p5_sc39959:120..1712         | p5_sc39959_gene_1                                  | p5.00_sc39959_p0001 |
| OPC26054 |       103 |       861 | -      | p5_sc40065 | OPN26054 | OPN26054 | augustus_masked-p5_sc40065-processed-gene-0.0          |        103 |       861 | maker
   | augustus_masked-p5_sc40065-processed-gene-0.0      | hypothetical protein SORBIDRAFT_10g005066
     | p5_sc40065:103..861          | augustus_masked-p5_sc40065-processed-gene-0.0      | p5.00_sc40065_p0001 |
| OPC26055 |      1229 |      1537 | +      | p5_sc40223 | OPN26055 | OPN26055 | snap_masked-p5_sc40223-processed-gene-0.2              |        884 |      1537 | maker
   | snap_masked-p5_sc40223-processed-gene-0.2          | 60s ribosomal protein l27a-3-like
     | p5_sc40223:884..1537         | snap_masked-p5_sc40223-processed-gene-0.2          | p5.00_sc40223_p0001 |
| OPC26056 |      1229 |      1847 | +      | p5_sc40223 | OPN26056 | OPN26056 | p5_sc40223_gene_1                                      |       1229 |      1873 | Fgenesh++
   | p5_sc40223_gene_1                                  | 60s ribosomal protein l27a-3-like
     | p5_sc40223:1229..1873        | p5_sc40223_gene_1                                  | p5.00_sc40223_p0002 |
| OPC26057 |       969 |      1610 | +      | p5_sc40236 | OPN26057 | OPN26057 | maker-p5_sc40236-exonerate_est2genome-gene-0.0         |        912 |      1677 | maker
   | maker-p5_sc40236-exonerate_est2genome-gene-0.0     | 60s ribosomal protein l27a-3-like
     | p5_sc40236:912..1677         | maker-p5_sc40236-exonerate_est2genome-gene-0.0     | p5.00_sc40236_p0001 |
| OPC26058 |       309 |      2661 | -      | p5_sc40253 | OPN26058 | OPN26058 | p5_sc40253_gene_1                                      |        149 |      2661 | Fgenesh++
   | p5_sc40253_gene_1                                  | Os02g0307900
     | p5_sc40253:149..2661         | p5_sc40253_gene_1                                  | p5.00_sc40253_p0001 |
| OPC26059 |       859 |      1728 | +      | p5_sc40286 | OPN26059 | OPN26059 | p5_sc40286_gene_1                                      |        646 |      2002 | Fgenesh++
   | p5_sc40286_gene_1                                  | sbp (s-ribonuclease binding protein) family protein
     | p5_sc40286:646..2002         | p5_sc40286_gene_1                                  | p5.00_sc40286_p0001 |
+----------+-----------+-----------+--------+------------+----------+----------+--------------------------------------------------------+------------+-----------+--------------
---+----------------------------------------------------+--------------------------------------------------------------------------------------------------------------------
-----+------------------------------+----------------------------------------------------+---------------------+
26059 rows in set (3.18 sec)
```

3) Scaffold Browser:

```
mysql> EXPLAIN select DISTINCT contig.contig_id, contig.assembly_ver, contig.contig_desc, contig.contig_length FROM contig JOIN cds ON contig.contig_id=cds.contig_id WH
ERE contig.contig_id LIKE 'p5_sc%' GROUP BY contig.contig_id ORDER BY CAST(MID(contig.contig_id,6) AS UNSIGNED);
+----+-------------+--------+--------+--------------------+--------------------+---------+---------------------------+-------+------------------------------------------
----+
| id | select_type | table  | type   | possible_keys      | key                | key_len | ref                       | rows  | Extra
    |
+----+-------------+--------+--------+--------------------+--------------------+---------+---------------------------+-------+------------------------------------------
----+
|  1 | SIMPLE      | cds    | index  | fk_cds_contig1_idx | fk_cds_contig1_idx | 32      | NULL                      | 26059 | Using index; Using temporary; Using files
ort |
|  1 | SIMPLE      | contig | eq_ref | PRIMARY,contig_id  | PRIMARY            | 32      | genemodelv2.cds.contig_id |     1 | Using where
    |
+----+-------------+--------+--------+--------------------+--------------------+---------+---------------------------+-------+------------------------------------------
----+
2 rows in set (0.00 sec)
```

Select statement:

```
mysql> select DISTINCT contig.contig_id, contig.assembly_ver, contig.contig_desc, contig.contig_length FROM contig JOIN cds ON contig.contig_id=cds.contig_id WHERE cont
ig.contig_id LIKE 'p5_sc%' GROUP BY contig.contig_id ORDER BY CAST(MID(contig.contig_id,6) AS UNSIGNED);
| p5_sc39251 | P5           | length=2028      |          2028 |
| p5_sc39303 | P5           | length=2027      |          2027 |
| p5_sc39316 | P5           | length=2030      |          2030 |
| p5_sc39476 | P5           | length=2024      |          2024 |
| p5_sc39541 | P5           | length=2475      |          2475 |
| p5_sc39543 | P5           | length=2021      |          2021 |
| p5_sc39629 | P5           | length=2993      |          2993 |
| p5_sc39702 | P5           | length=2017      |          2017 |
| p5_sc39758 | P5           | length=2017      |          2017 |
| p5_sc39834 | P5           | length=2014      |          2014 |
| p5_sc39857 | P5           | length=2014      |          2014 |
| p5_sc39875 | P5           | length=2014      |          2014 |
| p5_sc39959 | P5           | length=2011      |          2011 |
| p5_sc40065 | P5           | length=2010      |          2010 |
| p5_sc40223 | P5           | length=2007      |          2007 |
| p5_sc40236 | P5           | length=2004      |          2004 |
| p5_sc40253 | P5           | length=2884      |          2884 |
| p5_sc40286 | P5           | length=2002      |          2002 |
+------------+--------------+------------------+---------------+
2915 rows in set (0.28 sec)
```
