# Supplementary material for: PalmXplore: oil palm gene database
Source: Database (Oxford). 2018 Sep 18;2018:bay095. doi: 10.1093/database/bay095 (PMC6146135; doi:10.1093/database/bay095)
Supplement: Supplementary Data [file bay095_supp.zip › Supplementary_2.pdf]

The Oil palm gene Database

PALMXplore

Document : User Guide

Version : 3.0

Date : 1st July 2018

Advanced Biotechnology and Breeding Centre (ABBC)
Malaysian Palm Oil Board (MPOB)
No. 6, Persiaran Institusi, Bandar Baru Bangi,
43000 Kajang Selangor, MALAYSIA

PalmXplore DATA SUMMARY

	Total	Identifier
Pisifera5 (P5) Build Genome Scaffold	40,360	p5_sc*****<br>(e.g. p5_sc00001)
E. guineensis (EG5.1) Genome Build	16	Eg_Chr**<br>(e.g. Eg_Chr1)
Representative Gene Models	26,059	p5.00_sc*****_p****<br>(e.g. p5.00_sc00001_p00001)
Fgenesh++ Predicted Genes	27,915	p5.00_sc*****_p****<br>(e.g. p5.00_sc00001_p00001)
Seqping Predicted Genes	17,680	p5.00_sc*****_p****<br>(e.g. p5.00_sc00001_p00001)
Intronless Gene	3,658	EgIG_*<br>(e.g. EgIG_1)
Fatty Acid Biosynthesis (FAB) Genes	42	Eg*****_*<br>(e.g. EgACC_1)
Resistance (R) gene	210	Eg_rgh_***_*<br>(e.g. Eg_rgh_cnl_1)
Shell gene	1	SHELL
Virescens Gene	1	VIRESCENS
Mantled gene	1	MANTLED

About the current oil palm gene model set:

PalmXplore is a public-domain archive for the collection of high confident predicted oil palm (*Elaeis guineensis*) genes by Malaysian Palm Oil Board (MPOB). Our database presently contains 26,059 high confidence oil palm genes, generated by integrating the gene models of two gene-prediction pipelines: FGENESH++ developed by Softberry and Seqping by MPOB.

Quick Start

To access PalmXplore homepage, go to:

i. http://palmxplore.mpob.gov.my
ii. Alternatively, PalmXplore can be accessed via a direct link from Genomsawit portal (http://genomsawit.mpob.gov.my/).

PalmXplore HOME PAGE

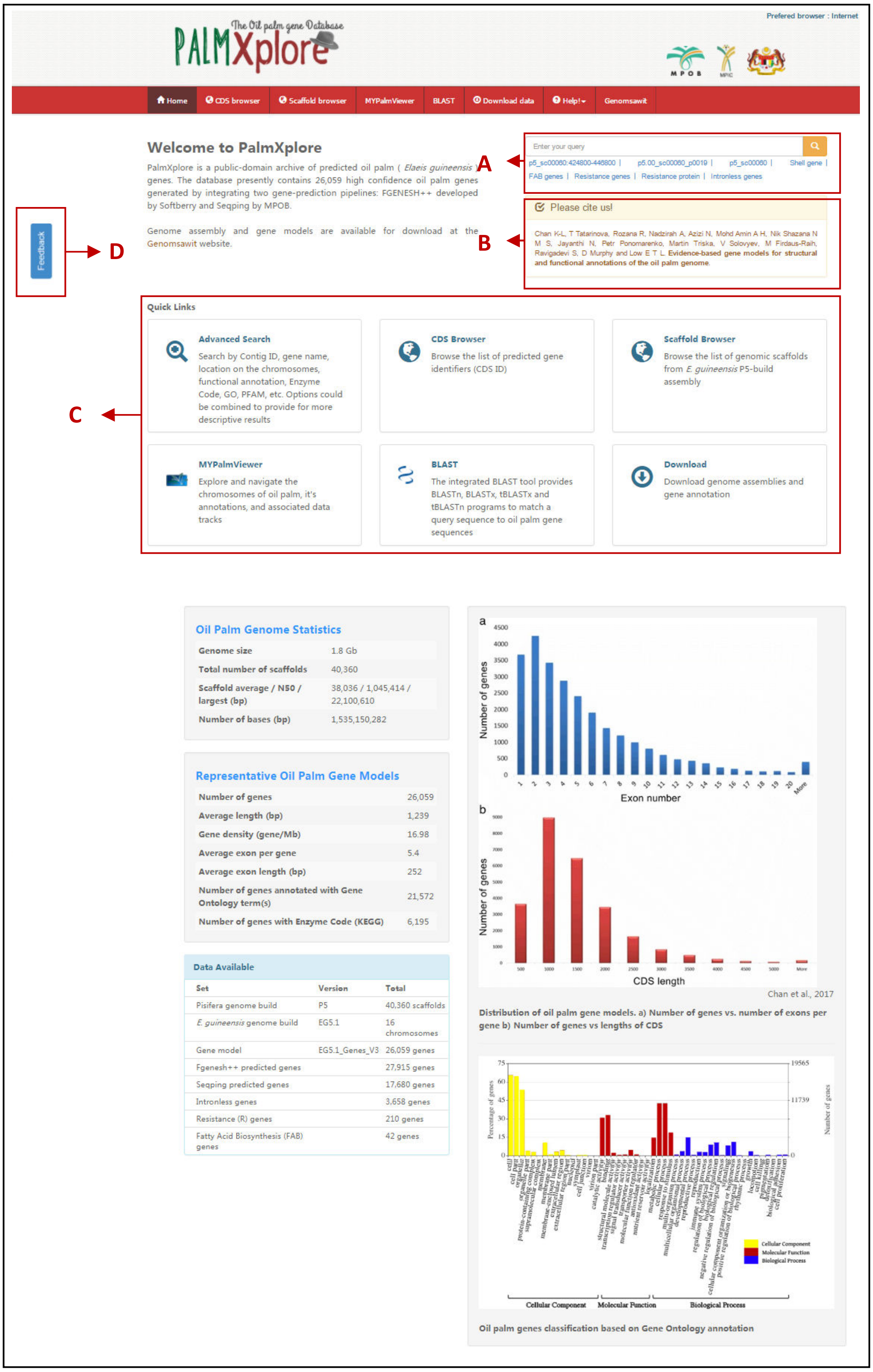

A: Quick Search

Genes may be searched by gene id, scaffold id, keywords, specific gene and/or location. Clickable examples with correct format are provided.
Search term examples include:

a. Location: p5_sc*****:******-******

The format for location search starts with the oil palm P5 scaffold id (example: p5_sc[00001 to 40360]), a colon (:), start and end location with a (-) separator.

Search term example(s): ***p5_sc00060:424800-446800***

b. Gene ID: p5.00_sc*****_p****

"p5.00" indicates the genome version, which is P5 build. "sc*****" indicates scaffold id. "p****", the digits after the "p" are auto-incrementally-generated by positions in the genome.

Search term example(s): ***p5.00_sc00060_p0019***

c. Scaffold ID: p5_sc*****

"p5" indicates the oil palm genome version, which is P5 build. "sc*****" indicates scaffold id.

Search term example(s): ***p5_sc00060***

d. Chromosome Number: Chr**

"Chr" indicates chromosome number oil palm.

Search term example(s): ***Chr1***
Eg_Chr1

e. Specific Gene(s):

i. Intronless gene: EgIG_*

"Eg" indicates species of oil palm (Elaeis guineensis). "IG" identifies the gene as intronless. Digit after "EgIG_" indicates intronless gene number.

Search term example(s): - ***EgIG_1***
- ***intronless gene***

ii. Fatty acid biosynthesis gene: Eg*****_*

"Eg" indicates species of oil palm (*Elaeis guineensis*). Letters after "Eg" indicates fatty acid gene name (e.g. ACC, CT, BCCP, BC, FABD, FABH, FABB, FABF,FABG, FABZ, FABI, FAB2, FAD2, FAD3, FATB or FATA). Digit after "_" indicates gene number.

Search term example(s): - ***EgACC_1***
- ***FAB genes***

iii. Resistance gene: Eg_rgh_***_*

"Eg" indicates species of oil palm (*Elaeis guineensis*). "rgh" identifies the gene as a resistance gene. Letters after "Eg_rgh_" indicates class of the resistance gene (e.g. cnl, kinase, mlo, rlk, rlp or others). Digit after "Eg_rgh_***_" indicates resistance gene number.

Search term example(s): - ***Eg_rgh_cnl_1***
- ***Resistance genes***

iv. Shell gene:

Search term example(s): - ***SHELL***
- ***shell gene***

v. Mantled gene:

Search term example(s): - ***EgDEF1***
- ***MANTLED***
- ***Mantled gene***

e. Other search terms:

i. Enzyme Code (EC)

Example(s): ***EC:3.5.1.98***

ii. GO ID

Example(s): ***GO:0016810***

iii. PFAM ID

Example(s): ***PF00850***

B: Please cite us!

Guide on how to cite PalmXplore system and its underlying publication(s).

C: Quick Links

a. Advanced Search tool:

Multiple options to select specific data types and parameters to formulate queries.

b. CDS Browser:

Thematic browser that lists all of the coding sequence (CDS) identifiers associated with the predicted oil palm genes.

c. **SCAFFOLD browser:**

Thematic browser that lists all of the genomic scaffolds from *E. guineensis* P5 build.

d. **MYPalmViewer:**

Explore and navigate the chromosomes of oil palm, annotation and associated data tracks

e. **BLAST tool:**

The integrated BLAST tool provides BLASTn, BLASTx, tBLASTx and tBLASTn programs to match a query sequence to oil palm gene sequences

f. **Download:**

List of data records of genome assemblies and gene annotation with downloadable FASTA and GFF3 (gene model information) files.

D: Feedback Form

Feedback form for questions, suggestions or issues found on PalmXplore system.

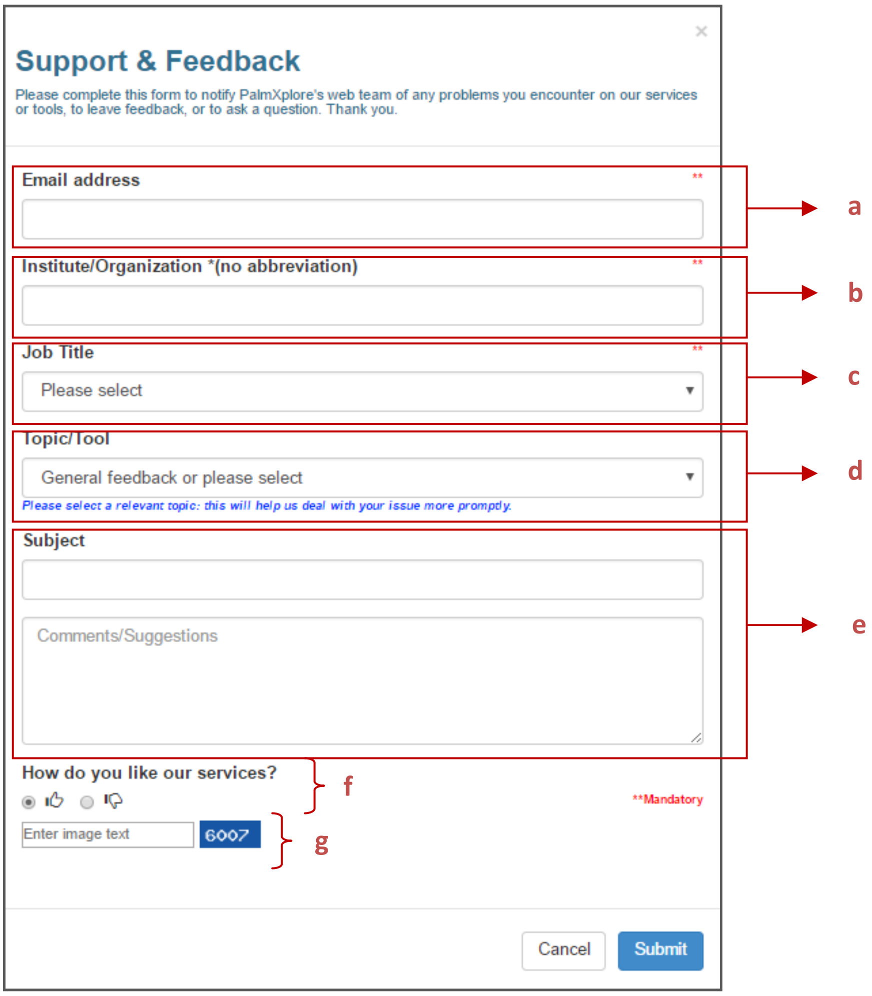

** indicates fields that are mandatory.

a. Email address

Please enter a valid email address.
Example: **nikshazana@mpob.gov.my**

b. Institute / Organization

Please enter full institute / organization name. Abbreviation is inadvisable.
Example: **Malaysian Palm Oil Board, Malaysia**

c. Job title

Please select your most relevant job title.

d. Topic/Tool

Please select a relevant topic, page or tool: this will help palmXplore's technical team deal with your issue more promptly.

e. Subject / Comments / Suggestions

Please enter relevant subject and provide any questions, comments, suggestions or issues found on PalmXplore system.

f. Satisfaction (How do you like our services)

Please make a selection on your satisfaction with services provided by PalmXplore.
: Like : Dislike

g. Captcha

Type the characters seen in the picture; if the characters can't be read submit the form and a new image will be generated.

ADVANCED SEARCH

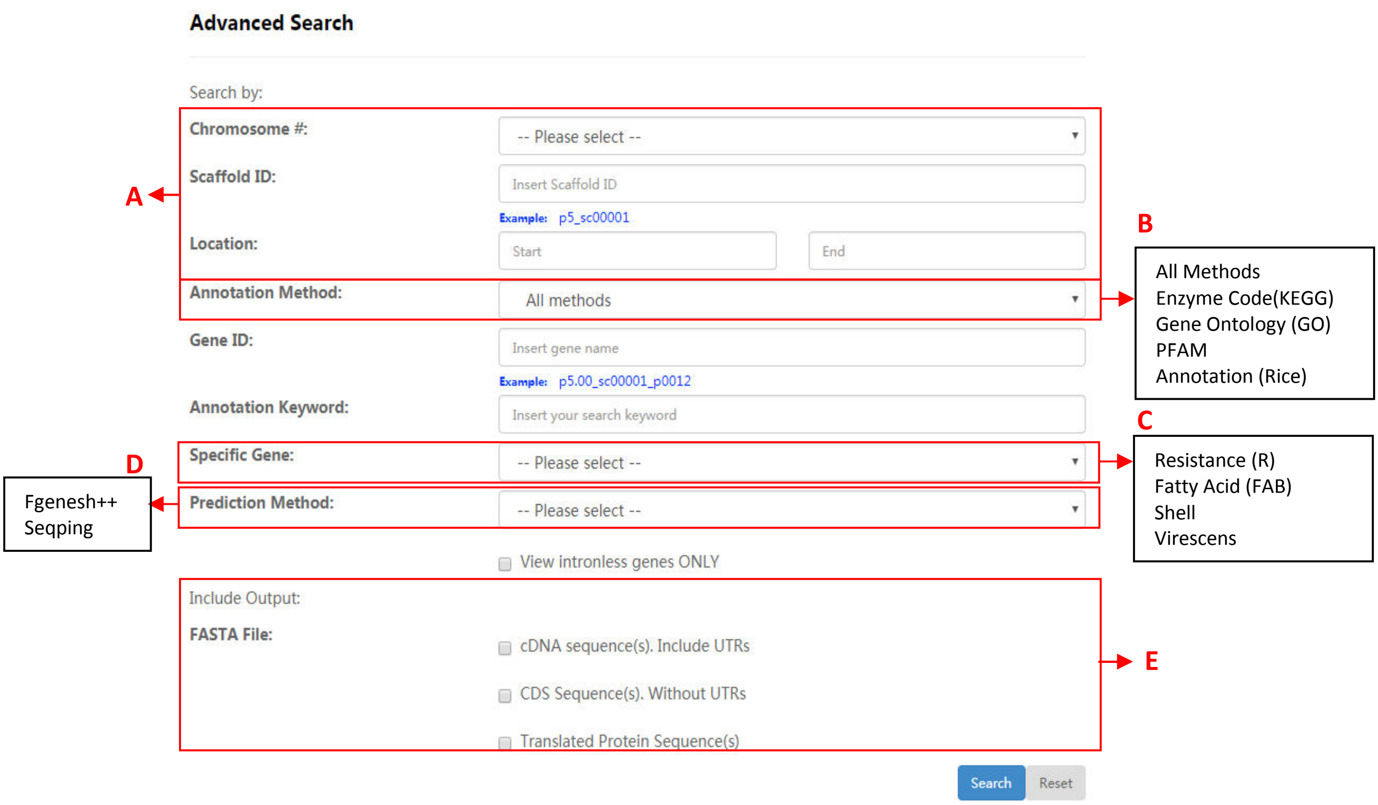

A: Chromosome Number, Scaffold ID and Location

Search genes within a genomic scaffold and location by providing Chromosome number (1 - 16) Scaffold ID and/or Start and End coordinates.

B: Annotation Method

Genes can be searched based on Enzyme Code (EC), GO, PFAM and rice annotation from BLAST.

i. **All methods**

Perform a cross search from Enzyme Code, GO and PFAM annotation results.

ii. **Enzyme Code (EC)**

Upon selecting this method, Enzyme Code (EC) input field is enabled.
*Search term example(s): **EC:3.5.1.98***

iii. **Gene Ontology (GO)**

Upon selecting this method, GO ID/GO term input field is enabled.
*Search term example(s): **GO:0016810***

iv. **PFAM**

Upon selecting this method, PFAM ID input field is enabled.
*Search term example(s): **PF00850***

v. Annotation from BLAST

Filter search according to the rice annotation from BLAST.

C: Specific Genes

Refine gene searching to specific genes, such as Resistance, Fatty Acid Biosynthesis, Shell, Mantled and Intronless.

D: **Prediction Method**

Refine gene searching according to prediction method; Seqping or Fgenesh++.

E: **Include Fasta File**

Include FASTA-formatted sequence file(s) (view/download) for:

a. cDNA sequence(s), which include UTRs
b. CDS Sequence(s), which does not include UTRs
c. Translated Protein Sequence(s)

REPORT PAGE

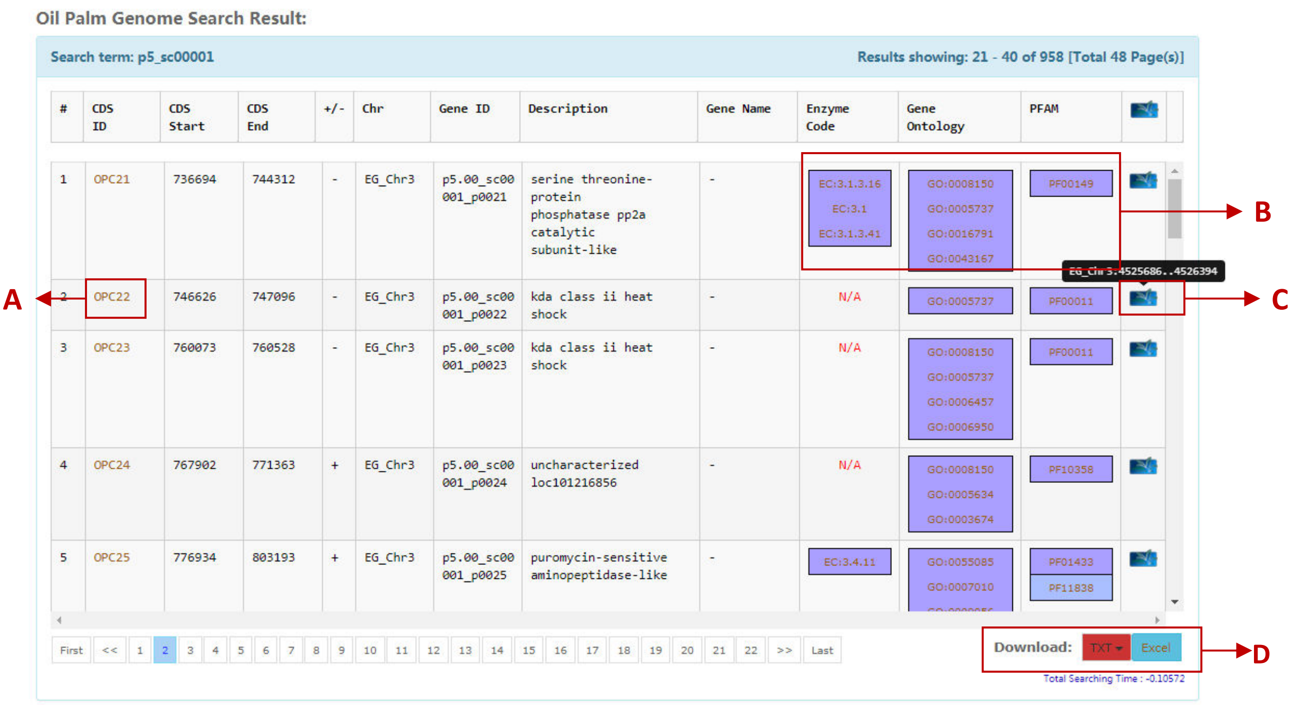

A: CDS Details

View details on the selected CDS

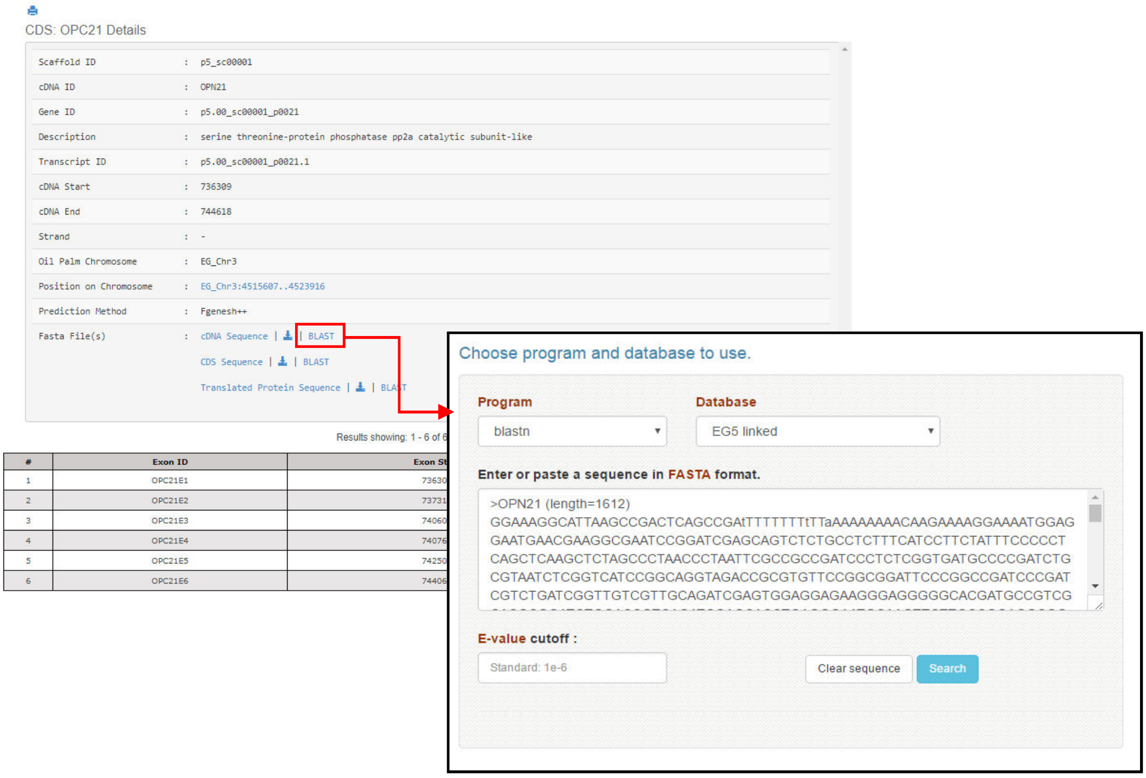

B: Enzyme Code (EC), GO and PFAM

View details of the selected CDS associated with functional annotation from Gene Ontology GO), PFAM and KEGG databases (including links to the external databases).

C: MyPalmViewer (GBrowse)

Click on the icon to open the oil palm genome browser (MyPalmViewer) displaying the associated gene region on the oil palm genome. The hits are visualized as features in the overview, region and details panels of the MyPalmViewer. Features in MyPalmViewer are hyperlinked to a page with additional information and sequences, as well as to external databases, where applicable.

D: Download

Click on "TXT" or "Excel" button to download the populated data from the query in tab-delimited or MS Excel format respectively. Downloading a large retrieval may take several minutes.

1) MyPalmViewer (GBrowse)

MYPalmViewer is accessible via :

i. http://gbrowse.mpob.gov.my/fgb2/gbrowse/Eg5_1/
ii. BLAST result

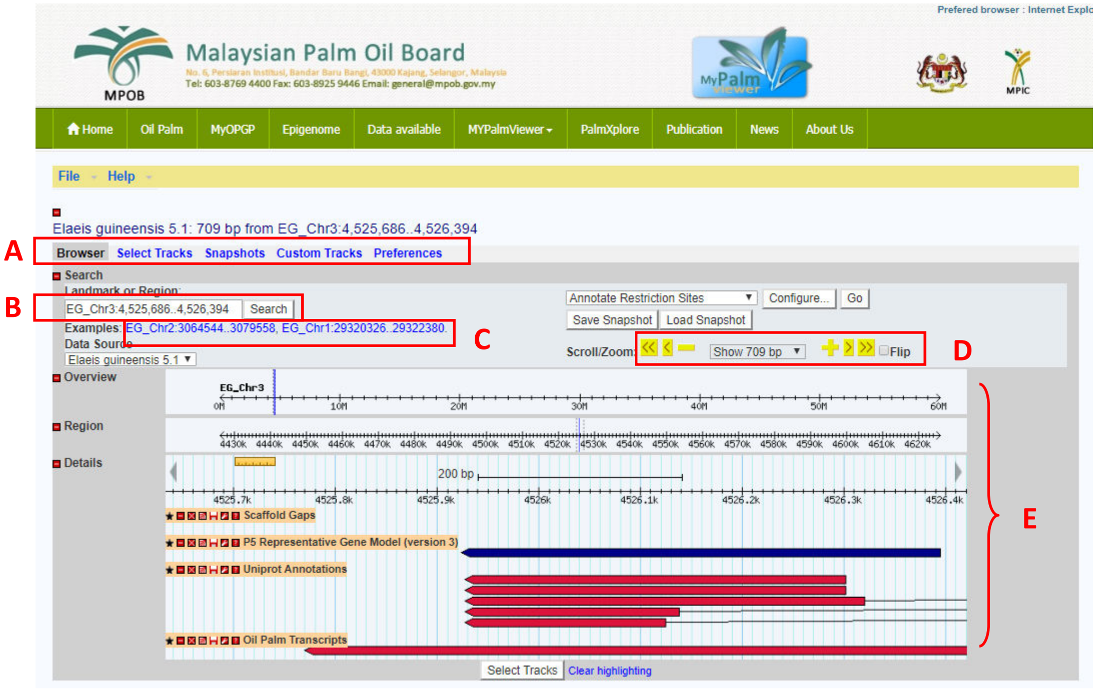

A: Gbrowse Main Menu

i. **Browser**:

Exhibit the region of the genome from the landmark searching. The region is displayed in three graphical panels:

a. Overview panel

This panel displays an entire assembled oil palm chromosome.

b. Region panel

This panel displays a portion of the genome surrounding the region of interest. It gives the context for the search region.

c. Detail panel

This panel displays a zoomed-in view of the genome corresponding to the overview's selection rectangle. The detail panel consists of one or more tracks showing annotations and other features that have been placed on the genome.

ii. **Select tracks:**

Tracks representing the mapped sequences to the oil palm genome. It allows users to select specific tracks in the browser.

iii. **Snapshots**:

Save the current browser screen as a picture

iv. **Custom tracks**:

It allows users to upload their own data set custom tracks.

v. **Preferences**:

User screen configuration setting of MYPalmViewer

B: Search by Landmark or Region

It allows user to search the browser based on keyword and location on the browser. The format for location search is start with the oil palm chromosome number (example: EG_Chr[1 to 16]), a colon (:), start location and end location with a double dot (..) separator.

Example: ***EG_Chr1:10..3000037***

C: Landmark or region input examples

D: Scroll and zoom options in MYPalmViewer

E: The selected tracks are visualize in section E

Available Tracks

As of July 2018, there are 12 categories of data tracks available in MYPalmViewer, which are listed below:

i. Oleifera 8 Scaffolds
ii. Pisifera 5 Scaffolds
iii. GeneThresher (TM) Sequences
iv. Oil Palm Transcripts
v. Oil Palm Protein Sequences
vi. P5 GlimmerHMM Gene Models
vii. Genetic Markers
viii. Uniprot Annotations
ix. Arabidopsis thaliana Genes
x. Oryza sativa Genes
xi. Oil Palm Retroelements
xii. Scaffold Gaps

** Also available are the DNA composition (GC Content) and restriction enzyme sites (Restriction Sites) of the P5-build.

Custom Tracks

Users can upload their custom tracks in six file format (BED, Gbrowse Feature File Format, GFF, GFF3, Wiggle (WIG) and BAM or SAM). Track files can also be fetched from URL.

2) BLAST Program

BLAST program is accessible via :

i. GenomSawit portal (http://genomsawit.mpob.gov.my)
ii. PalmXplore (http://palmxplore.mpob.gov.my)

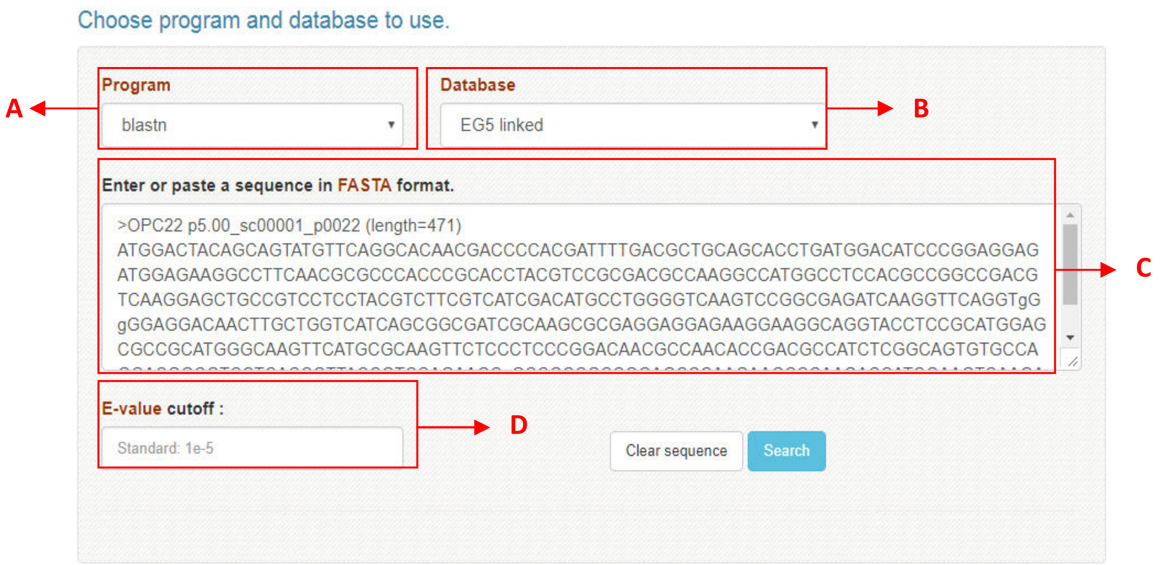

A: BLAST Program

Programs available for BLAST search

i. blastn

Compares a nucleotide query sequence against a nucleotide sequence database.

ii. blastp

Compares an amino acid query sequence against a protein sequence database

iii. blastx

Compares a nucleotide query sequence translated in all reading frames against a protein sequence database

iv. tblastn

Compares a protein query sequence against a nucleotide sequence database dynamically translated in all reading frames

v. tblastx

Compares the six-frame translations of a nucleotide query sequence against the six-frame translations of a nucleotide sequence database

B: Database

Databases available for BLAST search

i. EG5 linked (*E. guineensis* genome)

The file contains 16 EG5 chromosome (genetic scaffold) and 40,056 P5 scaffold sequences. The EG5 chromosomes resulted from a comparison of the P5-build to T128 and P2 genetic maps. The remaining P5 scaffolds that were not incorporated into the EG5 chromosomes were also included.

ii. O8 scaffolds (*E. oleifera* genome)

The file contains a scaffold sequences from the *E. oleifera* O8-build.

iii. P5 scaffolds (*E. guineensis* genome)

The file contains of scaffold sequences from the pisifera P5-build.

iv. *E. guineensis* transcriptome.fna

Thirty transcriptome libraries were constructed and sequenced using Roche 454, of which 22 were from *E. guineensis* and 8 from *E. oleifera*. The *E. guineensis* and *E. oleifera* libraries were assembled independently using Newbler.

v. *E. oleifera* transcriptome.fna

Thirty transcriptome libraries were constructed and sequenced using Roche 454, of which 22 were from *E. guineensis* and 8 from *E. oleifera*. The *E. guineensis* and *E. oleifera* libraries were assembled independently using Newbler.

vi. *E. guineensis* Genes.fna

A predicted nucleotide sequences (version 2) of pisifera P5-build that had similarity to known proteins in RefSeq.

vii. *E. guineensis* Genes.faa

A predicted amino acid sequences (version 2) of pisifera P5-build that had similarity to known proteins in RefSeq.

viii. BAC.fna

Genomic DNA from Dura palms were used to create the BACs. The BAC clones were pooled into 4 pools (BAC9, BAC10, BAC11 & BAC12) and sequenced using 454. Each BAC pool was assembled separately and manually finished.

ix. *E. guineensis* GT.faa

This is a contig and singlets of assembled reads from *E. guineensis* genomic libraries.

x. *E. oleifera* GT.faa

This is a contig and singlets of assembled reads from *E. oleifera* genomic libraries.

xi. Predicted Protein.faa (GT)

This is a nucleotide sequences of the predicted genes from the *E. guineensis* and *E. oleifera* contigs.

xii. Predicted Transcript.fna (GT)

This is an amino acid sequences of the predicted genes from the *E. guineensis* and *E. oleifera* contigs.

C: FASTA format

A sequence in FASTA format begins with a single-line description, followed by lines of sequence data. The description line is distinguished from the sequence data by a greater-than ">" symbol in the first column.

Example:

```
>Sequence
GGAGGGGGAAGCGGGTGCGGGAGACTAAATATATATTATATTTTTATAAGTTTGGTCTCTTCTCTC
GCTCTCCACCCTCTCTGTGATTTACGCGGCCATTTTTTTTCTCAGCGATATCAATAAAGAGGCGGT
AATCAACTAAAGGCGATGGGCGCGGGCGGACGAATGACGGCGAAAGAGCGGGAGGACCAGTCGGCG
ACGGCCCACGGCCCCGACGAGGACCCCTCCCTCCGGCGGTCGCCGACGGAGAAACCCCCATTCACA
TTGAGCCAGATCAAGAAGGCCATCCCCCCGCACTGCTTCCAGCGGTCCGTCCTCCGCTCCTTCTCC
TACGTCGTCCACGACCTCGTCATCTCCGCCGCCCTCTTCTACGTCGCCCTCGCCGTCATCCCGACC
CTCTCCCCGCCGCTCCTCCGCCTCGCCGCCTGGCCTCTCTACTGGGCCGCCCAGGGCTGCATCTTC
ACCGGCGTCTGGGTCATCGCCCACGAGTGCGGCCACCACGCCTTCTCCGACTCCTCCCTCCTCGAC
GACCTCGTCGGCCTCGTCCTCCACTCCGCCCTCCTCGTCCCCTACTTCTCCTGGAAGATCAGTCAC
CGCCGCCACCACTCCAACACCGGCTCGCTCGACCGCGACGAGGTCTTCGTCCCCAAGCGCAAGTCC
GCCCTCCCTTGGTACTCCAGGTACATCAACAACCCCCCGGGCCGCGTCCTCACCTTGGCGGTGACC
CTGATCCTCGGGTGGCCCCTGTACCTCGCCTTCAACGTCTCCGGCCGTCCGTATCCCCGGTTCGCC
TGCCACTACGACCCCTACGGCCCGATCTACTCGGACCGGGAGCGGGCCCAGATCTTCATCTCCGAC
GCGGGCGTTCTCGCGGCGTTCTACGCGCTGTGCCGGATCGC
```

D: E-value description

The statistical significance threshold for reporting matches against database sequences; the default value is 10, such that 10 matches are expected to be found merely by chance. If the statistical significance ascribed to a match is greater than the EXPECT threshold, the match will not be reported. Lower EXPECT thresholds are more stringent, leading to fewer chance matches being reported. Fractional values are acceptable.

-- *END of FILE* --
